# Discovery of 3-alkyl-5-aryl-1-pyrimidyl-1*H-*pyrazole derivatives as a novel selective inhibitor scaffold of JNK3

**DOI:** 10.1080/14756366.2019.1705294

**Published:** 2019-12-19

**Authors:** Youri Oh, Miyoung Jang, Hyunwook Cho, Songyi Yang, Daseul Im, Hyungwoo Moon, Jung-Mi Hah

**Affiliations:** College of Pharmacy and Institute of Pharmaceutical Science and Technology, Hanyang University, Ansan, Korea

**Keywords:** JNK, pyrazole, neurodegenerative diseases, SAR

## Abstract

3-alkyl-5-aryl-1-pyrimidyl-1*H*-pyrazole derivatives were designed and synthesised as selective inhibitors of JNK3, a target for the treatment of neurodegenerative diseases. Following previous studies, we have designed JNK3 inhibitors to reduce the molecular weight and successfully identified a lead compound that exhibits equipotent activity towards JNK3. Kinase profiling results also showed high selectivity for JNK3 among 38 kinases. Among the derivatives, the IC_50_ value of **8a,** (*R*)-2-(1-(2-((1-(cyclopropanecarbonyl)pyrrolidin-3-yl)amino)pyrimidin-4-yl)-5-(3,4-dichlorophenyl)-1*H*-pyrazol-3-yl)acetonitrile exhibited 227 nM, showing the highest inhibitory activity against JNK3.

## Introduction

c-Jun N-terminal Kinase (JNK) is a serine/threonine kinase that is one of the members of the mitogen-activated protein kinase (MAPK) family^1^. Activation occurs as a result of stimulation by factors such as oxidative stress, cytokines, and ultraviolet rays, thus inducing the apoptosis pathway of cells^2–6^. These JNKs have three isoforms. Among them, JNK1 and two are widely distributed in cells and tissues, but JNK3 is known to be distributed specifically in the brain^7^. These facts suggested that JNK3 may be a target for therapeutic agents for neurodegenerative diseases such as Alzheimer’s and Parkinson’s diseases^8–10^. Through many studies, it has been shown that inhibiting JNK3 suppresses the formation of beta amyloid, one of the causes of Alzheimer’s disease, and has proven its potential as a therapeutic target^11^. However, all three JNK isoforms have an ATP binding pocket with a highly conserved sequence; thus far very few drugs that exhibit only high selectivity for JNK3 have been discovered^1^. Due to the side effects that appear in response to these selectivity issues, there is increasing interest in research to find a JNK3-selective inhibitor.

**Figure 1.**
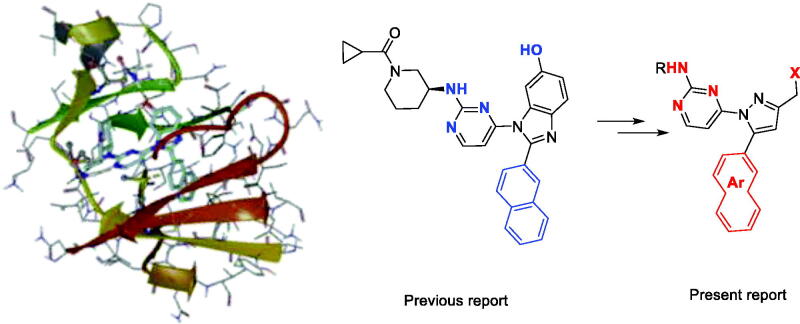
Docking structures of the previous JNK3 inhibitor (PDB: 3OY1) and design of the present 1-pyrimidyl-3-alkyl-5-aryl-1*H*-pyrazole scaffold.

We have found 1-heteroaryl-2-aryl-*1H*-benzimidazole derivatives that have selectivity for JNK3 through optimisation of a hit compound exhibiting JNK activity from our library in previous studies^12^. Based on the SAR results, we continued our efforts to design a new chemical scaffold of JNK3 inhibitors with reduced molecular weights for better Brain-Blood Barrier permeability^13^^,^^14^. During the development of new JNK3-selective inhibitors, we sought to maintain three interactions of the previous scaffold; *hydrogen bonds in the hinge region*, *hydrophobic interaction* of the aromatic ring, and the *hydrogen bond* of phenol in the benzimidazole scaffold, thus attempting to reduce the molecular weight. Therefore, we discarded benzene from the benzimidazole scaffold, retaining the hydrogen bond-possible moiety on 5-membered ring with final design and synthesis of the 3-alkyl-5-aryl-1-pyrimidyl-1*H*-pyrazole derivatives (Figure 1).

## Results and discussion

**Scheme 1.**
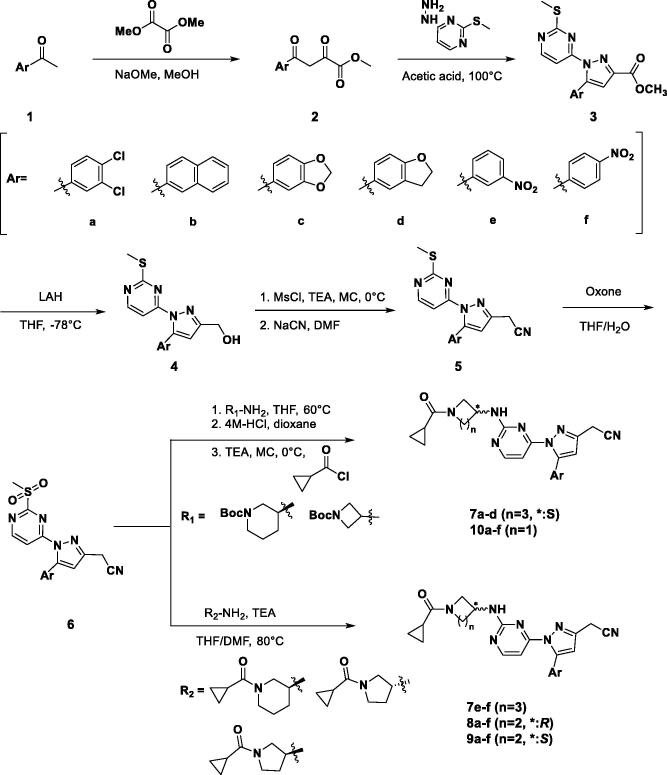
Synthesis of 3-alkyl-5-aryl-1-pyrimidyl-1*H*-pyrazole derivatives.

**Scheme 2.**
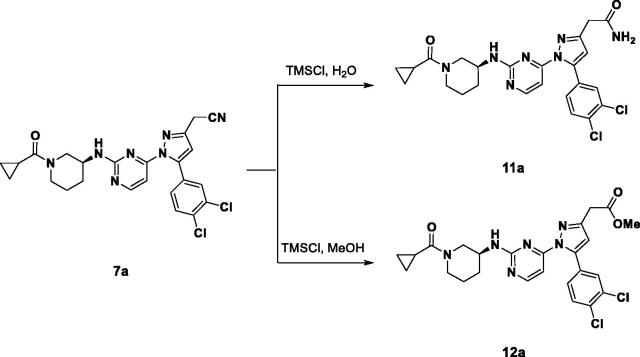
Synthesis of compound **11a** and **12a**.

The synthetic process of 3-alkyl-5-aryl-1-pyrimidyl-1*H*-pyrazole derivatives is shown in Scheme 1. We started with methyl ketones containing various aryl groups substituted (**1**) and formed enolate with sodium methoxide to react with dimethyl oxalate to produce beta ketone (**2**)^15^. The Knorr pyrazole synthesis was employed to form the pyrazole cores (**3**) using hydrazinyl pyrimidine and beta ketone^16^. After that, pyrazoyl ester was converted to alcohol (**4**) using lithium aluminium hydride^17^. The nitrile was introduced by the S_N_2 reaction with sodium cyanide following mesylation (**5**). Through the oxidation with potassium peroxymonosulfate of methyl sulphide to methylsulfone, a variety of amino groups were introduced to the pyrimidyl moiety (S_N_Ar)^18^^,^^19^; then the terminal amino group was deprotected and acylated to give the final products (**7a–d, 10a–f**). For compounds **7e–f**, **8a–f**, and **9a–f**, cyclopropylcarboxylated amine were directly incorporated. The terminal nitrile group was changed to an ester (**11a**) and carboxamide (**12a**) through Scheme 2. They were synthesised through hydrolysis of the **10a** performed at different conditions.

**Figure 2.**
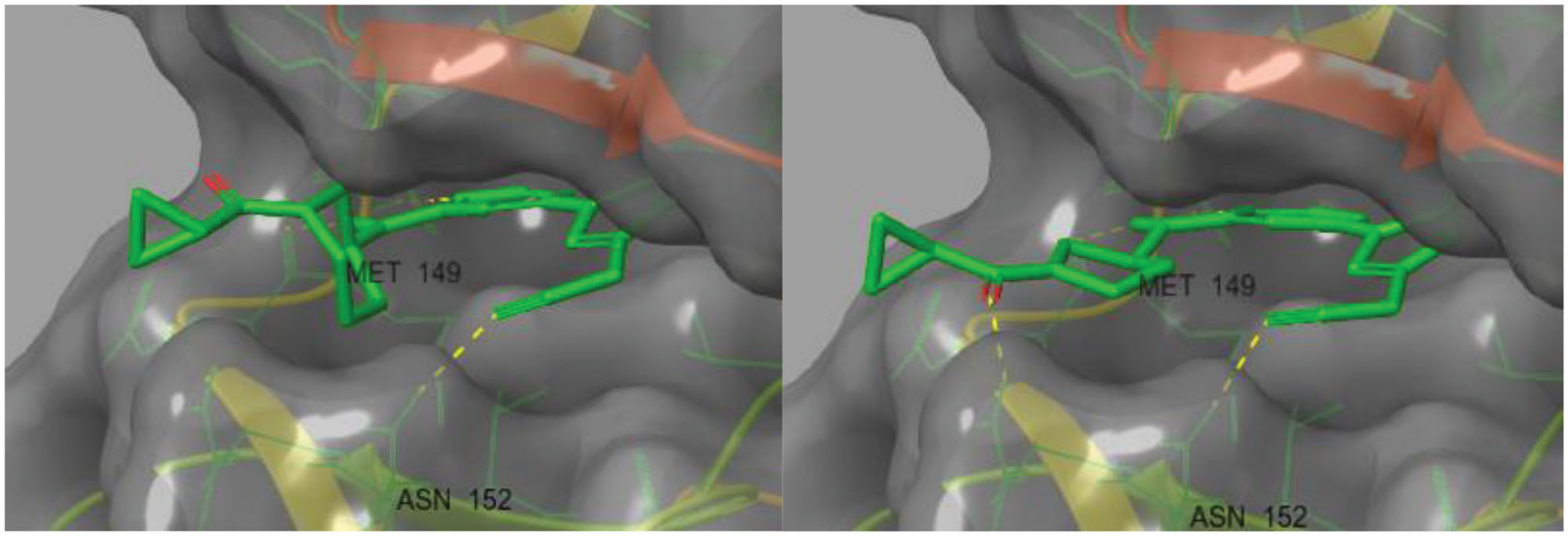
Comparison of docking structures of **7a** and **8a** at JNK3 (PDB: 3OY1).

All of the synthesised compounds, **7a**–**7f**, **8a**–**8f, 9a**–**9f,** and **10a**–**10f** were evaluated for their inhibitory activity against JNK3 (Table 1). First, we investigated the effect of the aryl group on their activity. The larger aryl groups such as the naphthyl and dichlorophenyl bound at position 5, elicited more potent activity towards JNK3 (**a, b vs. e, f**). This seems to be related to the electron density of the aromatic ring due to the sulfur-π interaction in the active site of JNK3. Compared to the mono-substituted phenyl groups, the relatively electron-rich dichlorophenyl and naphthyl groups could have formed a stronger π–π interaction, which may affect the activity. Next, to investigate the effect of the substituent at position 3, the compound **7a** was hydrolysed to convert it to an amide and a methyl ester (**11a, 12a**). As a result, the existing nitrile was the best in terms of potency, but not a noticeable difference. In an effort to reduce the molecular weight, the piperidine ring was diversified into pyrrolidine and azetidine with less carbon atoms. Surprisingly, when (*R*)-aminopyrrolidine was coupled to the pyrimidyl group instead of the (*S*)-aminopiperidine, the activities were increased approximately two to three fold (**7a vs 8a, 7b vs 8b, 7c vs 8c, 7d vs 8d**). This also suggested that the configuration of the amino group in the ring should be considered important for binding, even in the solvent exposure part for optimal extra-hydrogen bonding. The extra hydrogen bonding seemed more plausible in (*R*)-pyrrolidine (**8**) than in both cases of (*S*)-piperidine (**7**) and (*S*)-pyrrolidine (**9**) in the docking structures (Figure 2).

**Table 1.** Enzymatic activities of 1-heteroaryl-3-alkyl-5-aryl-1H-pyrazole derivatives. 
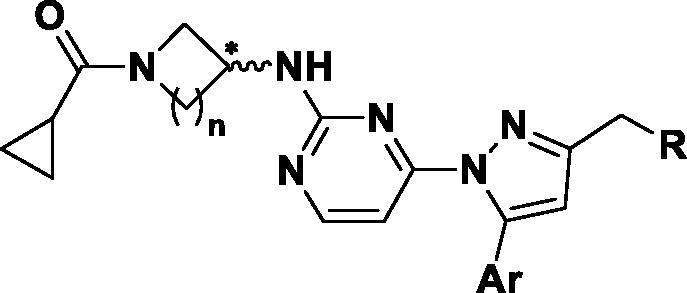

No	Ar	*n*	*(R/S)	R	JNK3 IC_50_ (μM)	No	Ar	*n*	*(R/S)	R	JNK3 IC_50_ (μM)
**7a**		3	*S*	CN	0.635	**7b**		3	*S*	CN	0.824
**8a**		2	*R*	CN	0.227	**8b**		2	*R*	CN	0.361
**9a**		2	*S*	CN	3.11	**9b**		2	*S*	CN	2.90
**10a**		1	*-*	CN	2.84	**10b**		1	*-*	CN	2.07
**11a**		3	*S*	CONH_2_	1.46						
**12a**		3	*S*	CO_2_Me	0.903						
**7c**		3	*S*	CN	4.60	**7d**		3	*S*	CN	7.90
**8c**		2	*R*	CN	2.18	**8d**		2	*R*	CN	4.42
**9c**		2	*S*	CN	8.57	**9d**		2	*S*	CN	3.25
**10c**		1	*-*	CN	5.58	**10d**		1	*-*	CN	NA
**7e**		3	*S*	CN	NA	**7f**		3	*S*	CN	>10
**8e**		2	*R*	CN	>10	**8f**		2	*R*	CN	7.89
**9e**		2	*S*	CN	NA	**9f**		2	*S*	CN	NA
**10e**		1	*-*	CN	NA	**10f**		1	*-*	CN	NA
Control compound					JNKI VIII^20^^,^^21^						0.005

Next, we used kinase panel screening in duplicate for compound **7a** over 38 different kinases at a single-dose concentration of 10 μM (Table 2). The compound was indeed a selective JNK3 inhibitor with an excellent selectivity profile. This compound has an inhibitory activity of 90% on JNK3 at a concentration of 10 μM; the inhibition activity was less than 20% for most other kinases except GSK3β, but for which, was also six fold selective in terms of IC_50_.

**Table 2.** Percentages of enzymatic inhibition exerted by **7a** (10 μM) on 38 selected protein kinases and enzymatic activities on selected protein kinases. 
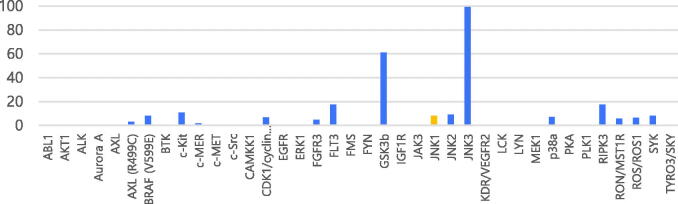

Compound IC_50_* (M):		IC_50_ (nM) Control Cmpd	Control Cmpd ID
Kinase:	**7a**		
GSK3β	3.96	2.30	Staurosporine
JNK3	0.635	5.13	JNKi VIII

We used Reaction Biology Corp. *Kinase HotSpot*^SM^ service (www.reactionbiology.com) for screening of **7a**.

**Figure 3.**
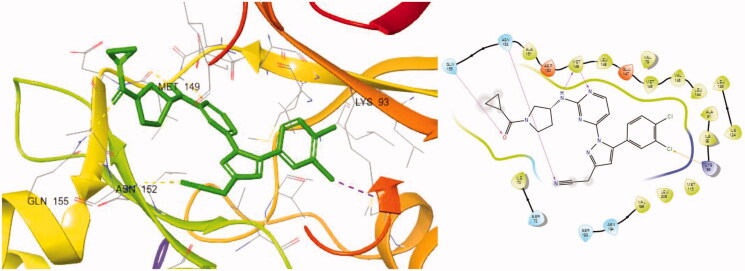
Docking structures of **8a** at JNK3 (PDB: 3OY1) and 2 D-interaction map.

A docking study was conducted to understand the binding mode of the novel JNK3 inhibitors (Figure 3). When we performed the docking experiment of **8a** with a known JNK3 structure (3OY1), it was shown that many of the interactions could contribute to complex stabilisation. First, the amino pyrimidine used as the hinge binder was found to form two hydrogen bonds with Met149 of JNK3 and another hydrogen bond is plausible between the oxygen of the cyclopropyl carboxamide group in **8a** and Gln155 in the extended hinge region. The third hydrogen bond seems possible between the nitrile located in the three position of pyrazole, which forms two hydrogen bonds between the backbone and the side chain of Asn152. Lastly, the aryl group at position 1 of pyrazole also fits into the hydrophobic pocket formed by residues such as Met148, Val79, Val145, Leu144, Ala91, Ile92, Ile124 and Leu128, especially noting that the dichlorophenyl ring could form a halogen bond with Lys93.

## Conclusions

We have successfully synthesised 3-alkyl-5-aryl-1-pyrimidyl-1*H*-pyrazole derivatives that were designed as novel JNK3 selective inhibitors in an effort to reduce the molecular weight from previous lead. Twenty-six compounds were synthesised and measured for their enzyme activity against JNK3. Particularly, compounds **7a, 7b, 8a,** and **8b** showed competitive activities against JNK3 with IC_50_ values of 0.635 μM, 0.824 μM, 0.227 μM, and 0.361 μM, respectively. Compound **7a** was, indeed, a selective JNK3 inhibitor with an excellent selectivity profile, especially compared to the activity towards similar protein kinases such as p38α, GSKβ, Erk, JNK1, and JNK2. We believe that this novel scaffold, 3-alkyl-5-aryl-1-pyrimidyl-1*H*-pyrazole will be highly useful in the development of JNK3 selective inhibitors, as therapeutic agents for neurodegenerative diseases.
